# Early Life Stress and Physical and Psychosocial Functioning in Late Adulthood

**DOI:** 10.1371/journal.pone.0069011

**Published:** 2013-07-05

**Authors:** Hanna Alastalo, Mikaela B. von Bonsdorff, Katri Räikkönen, Anu-Katriina Pesonen, Clive Osmond, David J. P. Barker, Kati Heinonen, Eero Kajantie, Johan G. Eriksson

**Affiliations:** 1 Department of Chronic Disease Prevention, National Institute for Health and Welfare, Helsinki, Finland; 2 Hjelt Institute,University of Helsinki, Helsinki, Finland; 3 Folkhälsan Research Center, Helsinki, Finland; 4 Gerontology Research Center and Department of Health Sciences, University of Jyväskylä, Jyväskylä, Finland; 5 Institute of Behavioural Sciences, University of Helsinki, Helsinki, Finland; 6 Department of General Practice and Primary Health Care, Institute of Clinical Medicine, University of Helsinki, Helsinki, Finland; 7 MRC Lifecourse Epidemiology Unit, Southampton, United Kingdom; 8 Chair of Fetal Programming, King Saud University, Riad, Saudi Arabia; 9 Hospital for Children and Adolescents, Helsinki University Central Hospital, Helsinki, Finland; 10 Unit of Primary Health Care, Helsinki University Central Hospital, Helsinki, Finland; 11 Vasa Central Hospital, Vaasa, Finland; University of Ottawa, Canada

## Abstract

**Background:**

Severe stress experienced in early life may have long-term effects on adult physiological and psychological health and well-being. We studied physical and psychosocial functioning in late adulthood in subjects separated temporarily from their parents in childhood during World War II.

**Methods:**

The 1803 participants belong to the Helsinki Birth Cohort Study, born 1934–44. Of them, 267 (14.8%) had been evacuated abroad in childhood during WWII and the remaining subjects served as controls. Physical and psychosocial functioning was assessed with the Short Form 36 scale (SF-36) between 2001 and 2004. A test for trends was based on linear regression. All analyses were adjusted for age at clinical examination, social class in childhood and adulthood, smoking, alcohol intake, physical activity, body mass index, cardiovascular disease and diabetes.

**Results:**

Physical functioning in late adulthood was lower among the separated men compared to non-separated men (*b* = −0.40, 95% confidence interval [95% CI]: −0.71 to −0.08). Those men separated in school age (>7 years) and who were separated for a duration over 2 years had the highest risk for lower physical functioning (*b* = −0.89, 95% CI: −1.58 to −0.20) and (*b* = −0.65, 95% CI: −1.25 to −0.05), respectively). Men separated for a duration over 2 years also had lower psychosocial functioning (*b* = −0.70, 95% CI: −1.35 to −0.06). These differences in physical and psychosocial functioning were not observed among women.

**Conclusion:**

Early life stress may increase the risk for impaired physical functioning in late adulthood among men. Timing and duration of the separation influenced the physical and psychosocial functioning in late adulthood.

## Introduction

Physical and mental health varies individually through the life course and decreases with age, which might cause decline in physical functioning and well-being later in life [1]. Health-related behaviours such as smoking and sedentary lifestyle as well as low educational attainment accelerate health decline in old age [2], [3]. However, decline in health in later life is not only related to biological and environmental factors occurring in adult life, but might also track back to early life experiences. Studies suggest that traumatic experiences in childhood such as childhood abuse, neglect, maltreatment and parental separation cause early life stress (ELS) and might serve as antecedents of health decline throughout the lifespan [4], [5]. Low childhood socioeconomic circumstances have also been associated with poor general health and physical function in later life [6]–[10]. A growing number of retrospective studies have reported that physical health and mental well-being in adulthood and at old age might stem from non-optimal growth and exceptional conditions during sensitive periods of growth and development early in life [4], [11]–[16].

Roughly 70 000 Finnish children experienced ELS in the form of temporary separation from their parents in two Finnish-Soviet wars during World War II (WWII). Children were evacuated to foster care in Sweden and Denmark. The Finnish Government carried out evacuations to protect the children from the strains of war. We have shown in our previous reports based on findings from the Helsinki Birth Cohort Study (HBCS) that the effect of war-time separation experience during childhood increased the risk for health problems later in life including hypertension, coronary heart disease (CHD) and type 2 diabetes [17]–[19], as well as depressive symptoms and hospitalisation for mental disorders [20], [21]. In the present study we hypothesize that, people who had experienced ELS have reduced physical and psychosocial functioning in later life. The current study investigates the differences in physical and psychosocial functioning in late adulthood according to war-time separation status as an indicator of stress experienced in early life. We use unique longitudinal data from HBCS in which a proportion of the participants had been separated from their parents during WWII.

## Materials and Methods

### Participants

The 1803 participants in this study belong to an epidemiological cohort, which includes 4630 men and 4130 women [22]. They were born in Helsinki University Central Hospital between 1934 and 1944, had visited child welfare clinics in the city and were still living in Finland in 1971, when a unique personal identification number was allocated to each member of the Finnish population. As previously described, we used random-number tables to select a subset of people who attended a clinical examination during 2001–2004. Of the 2902 invited subjects, 2003 men and women participated in the examination [23]. Of these, 189 subjects did not have reliable information on war time separation and because of this they were excluded from the analyses [24]. 1803 subjects had data on war time evacuation and physical and psychosocial functioning in late adulthood.

### Ethics Statement

The study protocol was approved by the ethical committee of the National Public Health Institute in Helsinki. Written informed consent was obtained from each participant before any study procedures were carried out.

### Early Life Stress (ELS)

Of the participants, 267 (14.8%) had been evacuated unaccompanied by their parents (“separated”) to Sweden or Denmark during WWII. Details of the historical context of the separations have been described elsewhere [25]. Data on evacuation were collected from a register in the Finnish National Archives, which gives full documentation of the children (n = 48628) evacuated by the Finnish Government. Information on age at and duration of the evacuation were obtained from the register. In addition, approximately 20000 children were evacuated abroad through personal contacts during WWII. These have not been officially registered. To identify these subjects, during the clinical survey in 2001–2004 we also collected information related to evacuation by asking the participants directly [20]. The remaining subjects (“non-separated”), who did not experience separation during WWII served as controls (n = 1536).

### Short Form 36 Physical and Psychosocial Functioning

Physical and psychosocial functioning was assessed with the physical component summary and the psychosocial component summary of the Finnish validated version of the RAND 36-Item Health Survey 1.0 (Short Form 36 [SF-36]) [26]–[29]. The physical component summary included four subscales: physical functioning, role limitations due to physical problems, pain and general health (Cronbach’s alpha = 0.80) and the psychosocial component summary included four subscales: role limitations due to emotional problems, energy, emotional well-being and social functioning (Cronbach’s alpha = 0.82). Each subscale included 2 to 10 items and item scores were summed and transformed to a scale from 0 to 100. Physical and psychosocial component summaries are continuous scales and range from 0 to 100, with a median score of 82.5 for the physical component summary and 87.3 for the psychosocial component summary. Higher scores imply better physical and psychosocial functioning.

### Socioeconomic Variables

Childhood socioeconomic status was evaluated based upon father’s highest occupational status, which was extracted from birth records, child welfare clinic and school health care records. Childhood socioeconomic status was categorized into three groups (senior clericals, junior clericals, manual workers) using a social classification originally devised by Statistics Finland [30]. Highest socioeconomic status in adulthood was based on register data from the Finnish Population Register Centre, which was obtained at 5-year intervals between 1970 and 1995. This was categorized into upper middle class, lower middle class, self-employed, and labourer.

### Confounding Variables

A clinical examination was performed by a team of 3 trained research nurses between 2001 and 2004, described in detail elsewhere [23]. Information on smoking, alcohol intake and physical activity were obtained from a questionnaire that the participants filled in during the clinical examination. Heights and weights were measured and the participants’ body mass index (BMI) was calculated (kg/m^2^). Cardiovascular disease (CVD) was defined as self-reported physician-diagnosed coronary heart disease and/or stroke. The participants came to the examination after an overnight fast and glucose tolerance was assessed using a 2-hour 75-g oral glucose tolerance test. The World Health Organization criteria for disturbances in glucose regulation were applied, in order to diagnose disturbances in glucose regulation [31].

### Statistical Analyses

Baseline differences for categorical variables were tested with logistic regression and continuous variables were compared by analysis of covariance (ANCOVA) adjusted for age and by the Mann-Whitney U test. The distributions of the physical and psychosocial component summaries were highly skewed. The rank transformation was used to normalize the distributions where SF-36 physical and psychosocial component summary scores divided into 6 equal sized gender-specific categories and they were coded with the numbers 1–6. The cut-off for the lowest category was 45.72 and highest 95.91 in men, and lowest 38.63 and highest 95.09 in women for the physical component summary, respectively. The cut-off for the lowest category in the psychosocial component summary was 53.57 and highest 97.48 in men, and lowest 43.58 and highest 96.34 in women, respectively. Differences in physical and psychosocial functioning between the separated and non-separated participants were investigated with multivariate linear regression. The analyses were first adjusted for age at clinical examination and highest social class in childhood and adulthood. Second, we added smoking, alcohol intake, physical activity and body mass index (BMI) to the model and finally CVD and diabetes. In addition, we tested whether age at separation and duration of separation was associated with physical and psychosocial functioning using the non-separated as controls. We split age at separation into 4 categories: infancy (<2 years); toddlerhood (2−4 years); early childhood (4−7 years) and school age (>7 years) and duration of separation into 3 categories: <1 years, 1 −2 years and >2 years which we have used in our previous studies [17], [19], [20]. Interaction between gender and separation status on physical functioning was *P* = 0.05 and on psychosocial functioning *P = *0.06, thus men and women were analysed separately. Statistical analyses were performed using SPSS (Statistical Package for Social Sciences) version 19.0 (SPSS, Armonk, NY, USA) for Windows.

## Results

Baseline differences according to separation status in childhood are presented in Table 1. Participants who had experienced separation from their parents were older than the non-separated. The separated had a significantly higher prevalence of CVD and diabetes compared to the non-separated, *P = *0.006 and *P* = 0.007, respectively. Those who had been separated during war time had lower childhood and adulthood socioeconomic status compared to the non-separated ones, *P* = 0.001 and *P* = 0.09, respectively. There were no significant differences in alcohol consumption, smoking, physical activity or BMI according to the separation status. The most common periods of separation were during toddlerhood and early childhood (mean = 4.8 years, SD = 2.4) and for approximately half of the separated participants, the duration was 1–2 years (mean = 1.7 years, SD = 1.0).

**Table 1:** Characteristics of the study cohort^a^.

	Separated	Non-separated	
	(n = 267)	(n = 1536)	
	Mean (SD) or %	Mean (SD) or %	*P*
Men (%)	50.2	46.9	0.18
Age at clinical examination (years)	63.7 (2.9)	60.9 (2.7)	*<0.0001*
Current smoker (%)	22.6	24.7	0.56
Weekly alcohol intake (%)	53.0	52.6	0.40
Exercise frequency ≥3 times/week (%)	49.4	42.5	0.33
BMI (kg/m^2^)	27.9 (4.4)	27.6 (4.8)	0.30
Prevalence of CVD (%)	15.1	7.8	*0.006*
Prevalence of diabetes (%)	21.4	15.3	*0.007*
Father’s highest social class in childhood (%)			*0.001*
Senior clericals	10.2	18.6	
Junior clericals	24.5	22.0	
Manual worker	65.3	59.3	
Highest social class in adulthood (%)			*0.09*
Upper middle	43.1	49.6	
Lower middle	39.0	37.7	
Self-employed	2.6	3.6	
Labourer	15.4	9.1	
Age at separation (years)^b^	4.8 (2.4)		
Infancy, <2 years (%)	9.9		
Toddlerhood, 2–4 years (%)	38.6		
Early childhood, 4–7 years (%)	28.8		
School age, >7 years (%)	22.7		
Duration of separation (years)^c^	1.7 (1.0)		
<1 year (%)	24.1		
1−2 years (%)	48.2		
>2 years (%)	27.6		

Abbreviation: BMI, body mass index.

a Categorical variables were tested with logistic regression and continuous variables were compared by the analysis of covariance (ANCOVA) and adjusted for age.

b Available for 87.0% of the separated.

c Available for 85.1% of the separated.

Table 2 shows mean scores for SF-36 physical and psychosocial functioning among the separated and non-separated participants. The scores in physical functioning, role limitations due to physical health and general health subscales were statistically significantly lower among the separated men compared to the non-separated (*P* = 0.005, *P* = 0.03 and *P* = 0.001, respectively). There were no differences in physical or psychosocial functioning according to war time separation status among women.

**Table 2:** Physical and psychosocial component subscales scores among separated (n = 267) and non-separated (n = 1536) participants^a^.

	ALL	Separated	Non-separated	
	Mean (SD)	Mean (SD)	Mean (SD)	*P*
**Physical component subscales**				
Physical functioning				
Men	85.7 (17.7)	82.5 (18.6)	86.3 (17.5)	*0.005* *
Women	79.1 (20.1)	77.6 (19.1)	79.4 (20.2)	0.07
Role limitations due to physical health				
Men	84.4 (29.5)	80.2 (31.7)	85.2 (29.0)	*0.03*
Women	77.3 (34.8)	77.3 (33.8)	77.3 (35.0)	0.68
Pain				
Men	82.9 (21.3)	80.0 (22.4)	83.4 (21.0)	0.06
Women	79.0 (22.0)	79.5 (20.4)	78.9 (22.3)	0.99
General health				
Men	62.8 (18.3)	58.5 (18.0)	63.6 (18.3)	*0.001* *
Women	62.3 (18.5)	62.1 (17.2)	62.4 (18.7)	0.70
**Psychosocial component subscales**				
Role limitations due to emotional problems				
Men	86.3 (28.2)	83.1 (30.8)	86.9 (27.6)	0.16
Women	80.5 (33.0)	80.5 (33.9)	80.5 (32.9)	0.76
Energy/fatigue				
Men	73.7 (18.8)	70.8 (19.8)	74.2 (18.5)	0.08
Women	67.4 (20.2)	67.9 (19.2)	67.3 (20.4)	0.94
Emotional well-being				
Men	83.1 (14.6)	81.1 (16.3)	83.5 (14.2)	0.14
Women	79.3 (15.7)	79.3 (15.0)	79.3 (15.8)	0. 73
Social functioning				
Men	91.2 (16.5)	89.9 (16.7)	91.4 (16.4)	0.24
Women	87.9 (19.1)	89.6 (17.8)	87.6 (19.3)	0.22

a Mann-Whitney U test.

* p-value <0.05 after Bonferroni correction.

Differences in SF-36 physical and psychosocial functioning according to gender among the separated and the non-separated are given in Table 3. Men who had been separated from their parents in childhood had an increased risk of decreased physical functioning in late adulthood (*b* = −0.45, 95% confidence interval [CI]: −0.78 to −0.13; *P* = 0.007). Adjusting the model further for smoking, alcohol intake, physical activity, BMI, CVD and diabetes did not change the results. Among men, the associations between the separation experience and psychosocial functioning in later life were similar and reached statistical significance also when adjusting the model further for smoking, alcohol intake, physical activity and body mass index (*b* = −0.37, 95% CI: −0.71 to −0.03; *P* = 0.03). Among women we did not find any associations between the separation experience and physical and psychosocial functioning. When the participants were further divided into two age groups (≤62 years and >62 years), the associations between the separation experience and physical and psychosocial functioning in later life remained the same.

**Table 3:** Differences (95% confidence intervals (CIs)) in SF-36 physical and psychosocial component summaries according to gender among participants who were separated in childhood compared to the non-separated*.

	Model 1		Model 2		Model 3	
	Differences (95%CI)	*P*	Differences (95%CI)	*P*	Differences (95%CI)	*P*
Physical component summary^a^						
Men	−0.45 (−0.78 to −0.13)	*0.007*	−0.46 (−0.78 to −0.14)	*0.004*	−0.40 (−0.71 to −0.08)	*0.014*
Women	0.16 (−0.17 to 0.49)	0.33	0.13 (−0.18 to 0.45)	0.41	0.20 (−0.11 to 0.52)	0.21
Psychosocial component summary^b^						
Men	−0.37 (−0.72 to −0.03)	0.03	−0.37 (−0.71 to −0.03)	0.03	−0.32 (−0.66 to 0.02)	0.06
Women	0.27 (−0.06 to 0.60)	0.11	0.27 (−0.07 to 0.60)	0.12	0.30 (−0.03 to 0.63)	0.08

Abbreviations: SF-36, short form 36.

Model 1 was adjusted for, age at the testing time, highest social class in childhood and adulthood.

Model 2 was adjusted for model 1+ smoking, alcohol intake, physical activity and body mass index.

Model 3 was adjusted for model 2+ presence of cardiovascular disease and diabetes.

a Models adjusted R square varied among men between 6% and 16% and among women between 2% and 11%.

b Models adjusted R square varied among men between 2% and 6% and among women between 1% and 3%.

Age at and duration of the separation also affected the outcomes studied (Table 4). The highest risk of lower physical functioning was observed among those men who were separated in school age compared to the non-separated participants (fully adjusted *b* = −0.89, 95% CI: −1.58 to −0.20; *P* = 0.01). Men who were separated for more than two years also had an increased risk of decreased physical functioning (fully adjusted *b* = −0.65, 95% CI: −1.25 to −0.05; *P* = 0.03). Among women, there were no differences in physical functioning according to age at or duration of separation. Table 5 shows that those men who were separated for more than two years were also at highest risk of lower psychosocial functioning compared with the non-separated (fully adjusted *b* = −0.70, 95% CI: −1.35 to −0.06; *P* = 0.03). Women who were separated for less than one year showed better psychosocial functioning than the non-separated participants (*b = *0.69, 95% CI: 0.02 to 1.35; *P* = 0.04). There were no differences in psychosocial functioning according to age at separation among the separated men and women compared with the non- separated.

**Table 4:** Differences (95% confidence intervals (CIs)) in SF-36 physical component according to age and duration of separation among the separated compared with the non-separated.

	Model 1		Model 2		Model 3	
	Differences (95%CI)	*P*	Differences (95%CI)	*P*	Differences (95%CI)	*P*
**Age at separation**						
**Men**						
Non-separated	Referent		Referent		Referent	
Infancy (<2y)	−0.52 (−1.55 to 0.51)	0.32	−0.72 (−1.71 to 0.27)	0.15	−0.74 (−1.71 to 0.23)	0.14
Toddlerhood (2–4y)	−0.28 (−0.78 to 0.22)	0.27	−0.26 (−0.75 to 0.23)	0.30	−0.20 (−0.68 to 0.28)	0.41
Early childhood (4–7y)	−0.20 (−0.83 to 0.42)	0.52	−0.20 (−0.81 to 0.41)	0.52	−0.15 (−0.75 to 0.45)	0.62
School age (≥7y)	−0.88 (−1.59 to −0.17)	*0.02*	−0.94 (−1.63 to −0.25)	*0.008*	−0.89 (−1.58 to −0.20)	*0.01*
**Women**						
Non-separated	Referent		Referent		Referent	
Infancy (<2y)	0.52 (−0.41 to 1.44)	0.27	0.44 (−0.46 to 1.34)	0.34	0.49 (−0.40 to1.38)	0.28
Toddlerhood (2–4y)	0.32 (−0.18 to 0.83)	0.21	0.26 (−0.23 to 0.75)	0.30	0.30 (−0.19 to 0.79)	0.23
Early childhood (4–7y)	0.17 (−0.42 to 0.75)	0.58	0.24 (−0.33 to 0.80)	0.41	0.28 (−0.28 to 0.84)	0.32
School age (≥7y)	−0.06 (−0.78 to 0.66)	0.87	−0.09 (−0.80 to 0.61)	0.79	0.02 (−0.70 to 0.73)	0.96
**Duration of separation (years)**						
**Men**						
Non-separated	Referent		Referent		Referent	
≤1	−0.52 (−1.16 to 0.12)	0.11	−0.46 (−1.09 to 0.17)	0.15	−0.38 (−1.00 to 0.24)	0.23
≤2	−0.24 (−0.71 to 0.24)	0.33	−0.26 (−0.72 to 0.21)	0.28	−0.25 (−0.71 to 0.21)	0.29
>2	−0.64 (−1.27 to −0.01)	*0.05*	−0.73 (−1.34 to −0.12)	*0.02*	−0.65 (−1.25 to −0.05)	*0.03*
**Women**						
Non-separated	Referent		Referent		Referent	
≤1	0.33 (−0.33 to 0.98)	0.33	0.26 (−0.39 to 0.90)	0.43	0.29 (−0.35 to 0.93)	0.37
≤2	0.36 (−0.12 to 0.83)	0.14	0.28 (−0.18 to 0.74)	0.24	0.33 (−0.13 to 0.79)	0.16
>2	−0.07 (−0.65 to 0.52)	0.82	0.04 (−0.52 to 0.61)	0.88	0.11 (−0.46 to 0.67)	0.71

**Table 5:** Differences (95% confidence intervals [CIs]) in SF-36 psychosocial component according to duration of separation (years) among the separated compared with the non-separated.

	Model 1		Model 2		Model 3	
	Differences (95%CI)	*P*	Differences (95%CI)	*P*	Differences (95%CI)	*P*
**Men**						
Non-separated	Referent		Referent		Referent	
≤1	0.13 (−0.54 to 0.79)	0.71	0.16 (−0.51 to 0.82)	0.64	0.21 (−0.45 to 0.88)	0.53
≤2	−0.41 (−0.90 to 0.08)	0.10	−0.38 (−0.88 to 0.11)	0.13	−0.40 (−0.90 to 0.09)	0.11
>2	−0.72 (−1.37 to −0.06)	*0.03*	−0.78 (−1.42 to −0.13)	*0.02*	−0.70 (−1.35 to −0.06)	*0.03*
**Women**						
Non-separated	Referent		Referent		Referent	
≤1	0.69 (0.02 to 1.35)	*0.04*	0.59 (−0.08 to 1.26)	0.08	0.60 (−0.07 to 1.27)	0.08
≤2	0.37 (−0.11 to 0.86)	0.13	0.33 (−0.15 to 0.80)	0.18	0.34 (−0.14 to 0.82)	0.17
>2	−0.07 (−0.65 to 0.52)	0.82	0.07 (−0.51 to 0.65)	0.81	0.12 (−0.47 to 0.72)	0.68

## Discussion

We studied the long term consequences of ELS on physical and mental functioning in later life in people who as children experienced temporary separation from their parents during WWII. Physical functioning differed between the separated and the non-separated men. Men separated in school age and separated for more than 2 years showed lower physical functioning compared to the non-separated. These associations were not seen among women. Duration of separation was also associated with psychosocial functioning, and those who had been separated for over 2 years had an increased risk of decreased psychosocial functioning when compared to the non-separated.

Our findings are consistent with other studies showing that ELS is associated with impaired physical and psychosocial functioning in later life [14], [15]. However, Edwards et al. have reported opposite results in relation to gender. They reported that men who had experienced childhood maltreatment had better physical function than women who had experienced maltreatment [32]. These differences may be due to the heterogeneity of ELS experiences. For example, we have shown that the children who were separated from their fathers because the fathers served in the military during war did not have a higher prevalence of depressive symptoms as adults (20). Other factors including socioeconomic status and health behaviours are also known to be associated with health [2], [9], [33], [34]. Both socioeconomic factors and health behaviours are important determinants of physical and mental functioning [9], [33], [35]. Some studies have shown, that physically active people with chronic diseases can maintain good physical functioning, supporting the importance of health behaviour [36], [37]. In the present study the separated group belonged to the lower socioeconomic classes compared with the non-separated group. No significant differences in physical activity were observed between the groups. We have shown that the separated groups had significantly higher prevalence of chronic diseases than the non-separated. Our findings were independent of age, social class, smoking, alcohol intake, physical activity, body mass index, cardiovascular disease and diabetes.

Our study design cannot disentangle the underlying mechanisms behind the associations between ELS and physical and psychosocial functioning in later life. There may be a large number of physiological, social and psychological explanations acting both individually and together. Furthermore, developmental differences between men and women may play a role. ELS research has focused on the developmental processes of physiological changes and re-setting of hormonal levels [38]. The modulations in physiological stress regulation may increase vulnerability to psychopathologies [39], [40]. ELS is known to influence the function of the hypothalamic-pituitary-adrenal axis (HPA) and consequently cortisol metabolism in adult life [25], [41], [42]. The stress experience and long term outcomes differ according to type of stress experienced, age at onset, duration but also gender seem to be an important factor. Gender is an important factor in disease vulnerability and there are sex-specific pathways to many mental and physical conditions, including coronary heart disease and aggressive behaviour, the prevalence of which is higher in men [43]. Gender differences may depend on the effects of sex hormones [44]. Gender differences may also depend on the stress reactivity of the HPA axis and the sympathetic nervous system, which can differ between the genders [45].

In this study, we used a longitudinal study design and well-characterized clinical data. We were able to use register data on socioeconomic status in childhood and adulthood. We also used reliable register data to define ELS and its timing. Despite this**,** we are aware that some children were evacuated via private routes and classified as non-separated [20]. The existence of the false controls would only diminish, not increase the observed group differences. The data on physical and mental functioning were self-reported based on the SF-36 form. The SF-36 has been found to be a valid and reliable health-related quality of life measurement in the Finnish population [26]. The present findings on physical and psychosocial functioning are comparable with the ones reported nationally. The limitations and possible selection bias of the Helsinki Birth Cohort Study have been discussed earlier [23]. Our study was restricted to people who had attended child welfare clinics, which were voluntary. Because of this the study could be unrepresentative of all people living in Helsinki. At that time, participation in child welfare clinics may have been related to families’ socioeconomic situations. However at the time of birth, social class distribution was similar to that in the city as whole. Likewise, there were exceptional nutrition and living conditions in Finland around the time of the Second World War, malnutrition was not uncommon and the evacuated children may have experienced better nutritional conditions. In addition to food shortages, families sent the children abroad for opportunity get to children safely away from aerial bombardments. These unusual background conditions, may limit the general application of our results.

Some other limitations of the study should also be noted. ELS may occur as a one-time exposure or it could include many adverse early experiences. Children who are in temporary foster care may have experienced at least two displacements, the first at the separation and the other upon returning home. In our study temporary separations took place during WWII and some of the separated children were separated two or more times during the war. The effect of ELS on adult physical and mental function can be expected to affect the severity and frequency of the adverse exposures, type of stress, quality of early life environment and timing of the separation [46], [47]. Unfortunately, we do not have information on the quality of foster care, which are likely to modulate the effect of ELS on adult physical and psychosocial functioning. Although early life stress was experienced in this historical cohort under exceptional circumstances, the developmental significance of stress in childhood is not limited to this study. Today early life stress caused by child maltreatment, poverty, immigration or war are worldwide phenomena and consequently little is still known about the long-term effects of these severe stress experiences on later life physical functioning.

In conclusion, the current study suggests that people who experienced ELS may be at increased risk for impaired physical functioning in late adulthood, this was observed at least for men. Timing and duration of the separation experience influenced both physical and psychosocial functioning in late adulthood. The long term effects of early life stress can differ largely from one individual to another depending on the individual coping mechanisms [48]. Men who experienced separation at school age and who were separated from their parents for more than two years are more vulnerable to the effects of early life stress. Therefore, future research should examine further gender differences in relation early life stress and take into account the cumulative effects of all life disadvantages.
